# Influence of Chemical Extraction Conditions on the Physicochemical and Functional Properties of Polysaccharide Gum from Durian (*Durio zibethinus*) Seed

**DOI:** 10.3390/molecules17066465

**Published:** 2012-05-29

**Authors:** Hamed Mirhosseini, Bahareh Tabatabaee Amid

**Affiliations:** Department of Food Technology, Faculty of Food Science and Technology, University Putra Malaysia, 43400 UPM Serdang, Selangor, Malaysia; Email: bahareh.ta@gmail.com

**Keywords:** seed gum, chemical extraction, solubility, water-holding capacity, oil-holding capacity

## Abstract

Durian seed is an agricultural biomass waste of durian fruit. It can be a natural plant source of non-starch polysaccharide gum with potential functional properties. The main goal of the present study was to investigate the effect of chemical extraction variables (*i.e*., the decolouring time, soaking temperature and soaking time) on the physicochemical properties of durian seed gum. The physicochemical and functional properties of chemically-extracted durian seed gum were assessed by determining the particle size and distribution, solubility and the water- and oil-holding capacity (WHC and OHC). The present work revealed that the soaking time should be considered as the most critical extraction variable affecting the physicochemical properties of crude durian seed gum.

## 1. Introduction

Polysaccharide gums are highly hydrophilic substances that are soluble or dispersible in water [1]. They can be used as thickeners, gelling agents, texture modifiers and stabilizers. Polysaccharide gums can be classified as food additives groups which adjust food quality in terms of stability, texture and appearance. There has been great interest in botanical sources of natural gums because plant polysaccharide gums represent one the most abundant raw materials in commercial liquid and semisolid foods [2]. Utilizing biomass from agricultural wastes as a raw material for valuable by-products has attracted the interest of researchers, especially in agriculture intensive countries [3]. On the other hand, the demand for a new potential source of plant gum has increased, because they are viewed as biodegradable natural polymers. Plant gums are certified as non-toxic and non-carcinogenic polymers with flexible applications in the food, cosmetic and pharmaceutical products. In fact, they have more advantages than other gums from animal and microbial sources due to their acceptance by consumers [4,5].

Durian (*Durio zibethinus* Murray) is the most popular seasonal fruit in South East Asia countries [3]. Only one-third of the durian is edible, whereas the seeds (20–25%) and the shell are usually thrown away [6]. Durian seed gum is a by-product from the durian industry. It could be exploited as a new plant-based hydrocolloid. There are many different methods for extraction of hydrocolloid from various plant sources. The effectiveness of different extraction conditions was studied by previous researchers [7,8]. Different solvents are used, depending on the matrix and its components. In many food processing operations, organic solvents are employed based on the polarity, solubility and mass transfer characteristics of the compounds to be extracted [9]. 

In the last decade, the physicochemical and functional characteristics of various plant gums such as locust bean gum [10], mesquite seed gum [11], basil seed gum [12], karaya gum [13], gum Arabic, pectin and carboxymethyl cellulose (CMC) [14,15,16,17,18,19] have been studied by several researchers. To the best of our knowledge, the effect of chemical extraction on physicochemical properties of durian seed gum has not been reported yet. The aim of the present study was to investigate the effect of chemical extraction conditions on the physicochemical properties of durian seed gum. The chemical extraction variables studied were the decolouring time (60–180 min), soaking temperature (25–55 °C) and soaking time (4–12 h). It should be noted that the influence of aqueous extraction on functional properties of durian seed gum was reported in our previous study [20]. 

## 2. Results and Discussion

### 2.1. Volumes-Weighted Mean (D [4,3])

The volume-weighted mean (D [4,3], average particle size) of durian seed gum in the aqueous system varied from 43.5 to 135.8 μm, depending on the extraction condition. The durian seed gum with smaller particle size might be preferred, because the hydration rate could be increased under such conditions. Table 1 showed that the volume-weighted mean (Y_1_) was positively proportional to the single effect of all extraction variables. Conversely, it was negatively affected by the interaction effects of the soaking temperature with the decolouring time and soaking time (Table 1). Table 2 indicated that the soaking temperature and decolouring time showed the most and least significant (*p* < 0.05) effects on the volume-weighted mean, respectively. 

**Table 1:** Regression coefficients, *R*^2^ and p-value of lack of fit for final reduced models.

Regression coefficient	Volume mean (μm)	Span	Solubility (80 °C, %)	WHC (g water/100 g gum)	OHC (g oil/100 g gum)
Constant	−51.76	4.2821	20.4917	279.50	185.18
b_1_	0.82	−0.0021	0.2796	-	0.25
b_2_	12.68	−0.7422	-	−16.72	24.24
b_3_	1.22	0.0848	−0.0744	−3.90	−7.32
b_1_^2^	-	-	-	-	−0.00
b_2_^2^	-	0.0243	-	-	−0.66
b_3_^2^	0.05	-	0.0107	-	0.06
b_12_	-	0.0022	-	-	−0.05
b_13_	−0.02	−0.0005	−0.0063	-	0.01
b_23_	−0.39	-	-	0.50	−0.15
R^2^	0.750	0.824	0.925	0.780	0.979
*p*-value	0.027 *	0.011 *	0.027 *	0.038 *	0.004 *
Lack of fit (*p*-value)	0.188	0.499	0.236	0.353	0.508

b_i,_ b_ii_ and b_ij_: the estimated regression coefficients for the main linear effects, quadratic effects and interaction effects, respectively; 1-decouloring time; 2-soaking time; 3-soaking temperature); OHC: Oil-holding capacity; WHC: Water-holding capacity; *: significant (*p* < 0.05).

**Table 2:** *p*-Value and F-ratio of chemical extraction variables in final reduced models.

Variables		Main effects			Quadratic effects			Interaction effects		
		*x*_1_	*x*_2_	*x*_3_	*x*_11_	*x*_22_	*x*_33_	*x*_1_ *x*_2_	*x*_1_ *x*_3_	*x*_2_ *x*_3_
Volume Weighted mean (μm)	*p*-value	0.020	0.024	0.557 *	-	-	0.021	-	0.042	0.014
	F-ratio	7.919	7.328	0.372	-	-	7.795	-	5.636	9.272
Span	*p*-value	0.853 *	0.003	0.013	-	0.034	-	0.023	0.046	-
	F-ratio	0.036	15.674	9.400	-	6.215	-	7.513	5.373	-
Solubility (80 °C, %)	*p*-value	0.011	-	0.859 *	-	-	0.042	-	0.010	-
	F-ratio	9.200	-	0.033	-	-	5.303	-	9.600	-
WHC	*p*-value	-	0.014	0.011	-	-	-	-	-	0.007
	F-ratio	-	8.433	9.223	-	-	-	-	-	10.857
OHC	*p*-value	0.243 *	0.002	0.002	0.005	0.002	0.005	0.017	0.011	0.034
	F-ratio	1.753	38.267	35.557	22.591	31.629	22.307	12.215	15.288	8.346

* Non-significant at *p* > 0.05; OHC: Oil-holding capacity (g oil/100 g gum); WHC: Water-holding capacity (g water/100 g gum); *x*_1_, *x*_2_ and *x*_3_ represents the main or single effect of the decolouring time, soaking time and soaking temperature, respectively. *x*_1_^2^, *x*_2_^2^ and *x*_3_^2^ represents the quadratic effect of the decolouring time, soaking time and soaking temperature, respectively. *x*_1_ *x*_2_, *x*_1_ *x*_3_ and *x*_2_ *x*_3_ represent the interaction between the decolouring time and soaking time, interaction between the decolouring time and soaking time and interaction between soaking time and soaking time, respectively.

**Figure 1:**
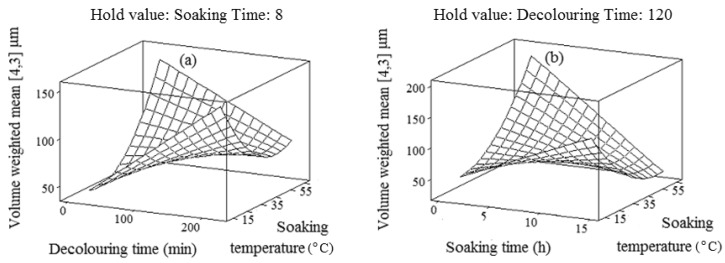
Response surface plots for explaining the variation of volume-weighted mean as a function of (**a**) decolouring time and soaking temperature and (**b**) soaking time and soaking temperature.

As shown in Figure 1a,b, the particle size increased with prolonged decolouring time and soaking time. This could be explained by the fact that decolouring or soaking for the long time may lead to the passage of large macromolecules (e.g., polysaccharides) and insoluble matters into the final extract. Hence, this phenomenon is probably responsible for increasing the volume-weighted mean (or large particle size). The results indicated that the main and quadratic terms of the soaking temperature were positively related to the volume-weighted mean; while the interaction effects of the soaking temperature with the decolouring time and soaking time were negatively proportional to the volume-weighted mean. The increase in the soaking temperature up to a certain level led to decrease volume-weighted mean. However, a further increase in the soaking temperature than the certain level resulted in larger volume-weighted mean (Figure 1a,b). This observation highlighted that the volume-weighted mean was more significantly influenced by the interaction effect of the soaking temperature with other extraction variables rather than its single effect. The higher volume-weighted mean (or larger particle size) might not be preferred, because the hydration process could be longer under such conditions.

### 2.2. Span or Particle Size Distribution

The width of the size distribution is determined by measuring the span. It is defined as Span = [D(0.9) − D(0.1)]/D(0.5); where D (0.10), D (0.50) and D (0.90) are the particle diameters at 10% (small particles), 50% (medium size particle) and 90% (large coarse particle), respectively (Equation 2). The smaller span represents the narrower size distribution [21,22]. A very high span with a large D (0.90) is not desirable because it is related to the presence of a considerably higher volume of coarse particles (D, 90) [22,23]. According to Equation (2), the span becomes larger when D (0.90) is larger or D (0.10), and D (0.50) are smaller. In fact, the presence of a higher volume of large coarse particles (D, 0.90) or lower volume of medium (D, 0.50) and small (D, 0.10) particles results in larger span (or wide particle size distribution). Conversely, the smaller span (or narrower particle size distribution) may be due the larger volume of the medium (D, 0.50) and small (D, 0.10) particles [22]. 

**Figure 2:**
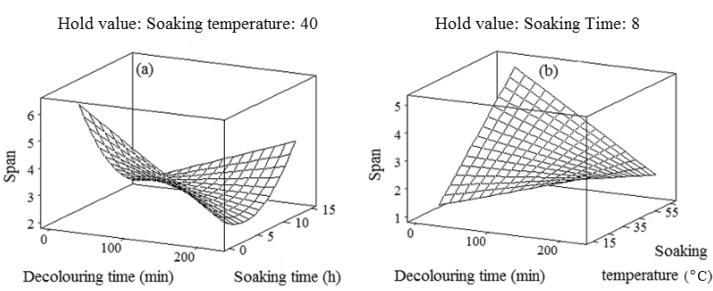
Response surface plots for explaining the variation of span as a function of (**a**) decolouring time and soaking time and (**b**) decolouring time and soaking temperature.

Table 1 showed that the span (Y_2_) was negatively associated with the single effects of the decolouring time and soaking time. In addition, the interaction effect of the decolouring time and soaking temperature negatively affected the span. Conversely, the interaction effect of the decolouring time and soaking time exhibited a significant (*p* < 0.05) positive effect on the span (Table 1). The results showed that the decolouring time and soaking time had the least and most significant (*p* < 0.05) effect on the span or particle size distribution, respectively (Table 1). The curves shown in Figure 2a confirmed the nonlinear relationship between the chemical extraction variables and span (Y_2_). This could be explained by the fact that the span was more significantly (*p* < 0.05) influenced by the quadratic and interaction effects of extraction variables rather than their single effects. The span increased not only by prolonging the decolouring time, but also by extending the soaking time (Figure 2a). Ideally a low span with a small D (0.90), moderate D (0.50) and comparatively large D (0.90) is the suitable physical property in the crude gum. As also reported by Gorji *et al*. [24], a large D (0.9) and high span indicated that particle size distribution was significantly influenced by the presence of large particles. 

### 2.3. Solubility

Some hydrocolloids induce their maximum functional properties after fully dissolving in water [25]. Full solubility is also favorable from the viewpoint of the appearance and texture. The interaction of hydrocolloids with water molecules can reduce the diffusion rate of water molecules into the hydrocolloid matrix. The interaction rate depends on the hydrogen bonding and temperature as well as the formation of water clusters [20]. As reported by previous researchers [25,26], a heating process was required to totally dissolve some of the hydrocolloids in order to induce the full viscosity. However, the heating process should not be very high because the viscosity might be decreased due to thermal hydrolysis of the hydrocolloid [25]. The results indicated that crude durian seed gum provided a relatively medium solubility in the elevated temperature (21.4–53.2%). 

**Figure 3:**
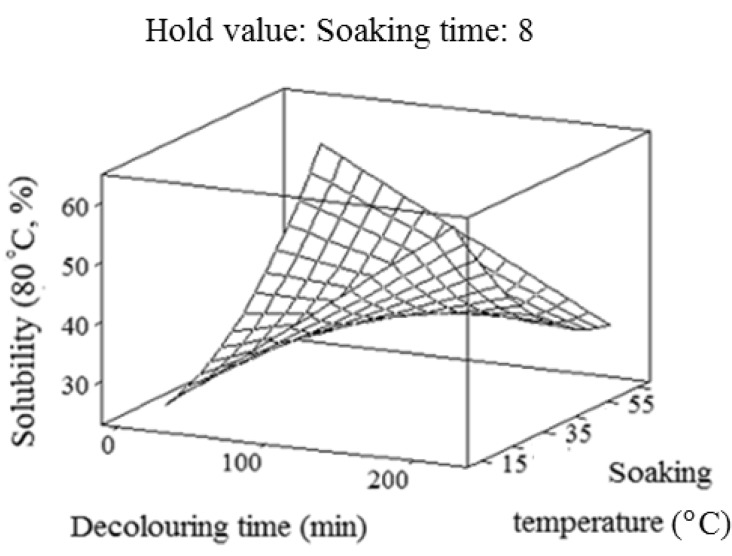
Response surface plot for explaining the variation of solubility (80 °C, 30 min) as a function of decolouring time and soaking temperature.

A crude gum may not be fully dissolved in water due to the presence of insoluble particles and impurities. The water solubility depends on the presence of hydroxyl groups, nature of monosaccharide, inter sugar linkages (α or β) and the ability to associate by intermolecular interactions [24]. The relatively wide range of solubility could be due to the different particle size (43.5 to 135.8 μm) of crude gums extracted under different extraction conditions. The particle size is a critical factor affecting the solubility [25]. The larger and coarser particles take longer time to be dissolved due to the longer time required for the penetration of water into the sample matrix. Conversely, the presence of smaller particle leads to shorter time for the penetration of water into the sample matrix, thus inducing better solubility [25]. Koocheki *et al*. [27] also reported a relative low solubility (25%) for *Lepidium perfoliatum* seed gum. They described that the low solubility of *Lepidium perfoliatum* seed gum might be related to the presence of impurities in the chemical structure of the crude gum. In the current study, the low solubility of the crude gum could be related to the considerable impact of the soaking process on the chemical composition of durian seed gum. Figure 3 showed that the solubility (80 °C) increased with prolonging the decolouring time. Conversely, the solubility (80 °C) decreased when the soaking temperature was increased (Figure 3). This could be related to the significant (*p* < 0.05) effect of the soaking temperature on the chemical composition of the seed gum. 

The dissolution process can be endothermic or exothermic, depending on the amount of energy required for the process. When water dissolves a substance, the water molecules attract or bond to the particles (molecules or ions) of the substance, thereby separating the particles from each other. This involves strong bonds between the substance particles and water molecules caused by water’s polarity. It requires energy to fracture the bonds between the molecules or ions of the solute. When water molecules bond to the solute molecules or ions, energy will be released. If the energy required to separate the particles of the solute is more than the energy released by the water molecules bonded to the particles, the dissolution process is an endothermic reaction. If the energy required for separating the particles of the solute is less than the energy released by the water molecules bonded to the particles, the dissolution process is an exothermic reaction [28]. 

The results indicated that the decolouring time exhibited the most significant (*p* < 0.05) effect on the solubility (80 °C, Y_3_); while the soaking temperature had the least significant (*p* < 0.05) effect on the solubility (80 °C, Y_3_) (Table 2). This observation can be explained by the fact that the increase in the soaking temperature may lead to release of the insoluble polysaccharide (e.g., insoluble dietary fiber) or other insoluble impurities into the final extract, thereby reducing the solubility. On the other hand, Table 2 shows that the soaking time did not show any significant (*p* > 0.05) effect on the solubility (80 °C) of crude durian seed gum. Therefore, it should not be kept in the final reduced model fitted to the solubility data (Table 1).

### 2.4. Water-Holding Capacity (WHC)

Hydration properties are influenced by the swelling capacity, solubility, and water-holding capacity (WHC) [29]. WHC represents the ability of a substance to associate with water under limited water conditions [30]. The wide industrial application of gums is due to their ability of holding water to produce gels or highly viscous solutions [31]. Plant gums are solid materials usually consisting of polysaccharides, which are either water-soluble or absorb water and swell up to form a gel; while they are insoluble in oils or organic solvents [32]. WHC represents the percentage of hydrophilic fraction, which has a greater affinity to absorb water [33]. It depends on pore size, conformational structure and capillary of the molecule, which are strongly correlated with the extent of molecule hydration with polar compounds along with the hydrophilic interaction through hydrogen bonding [34]. Therefore, high WHC is associated with the enhancement of the hydrophilic character.

**Figure 4:**
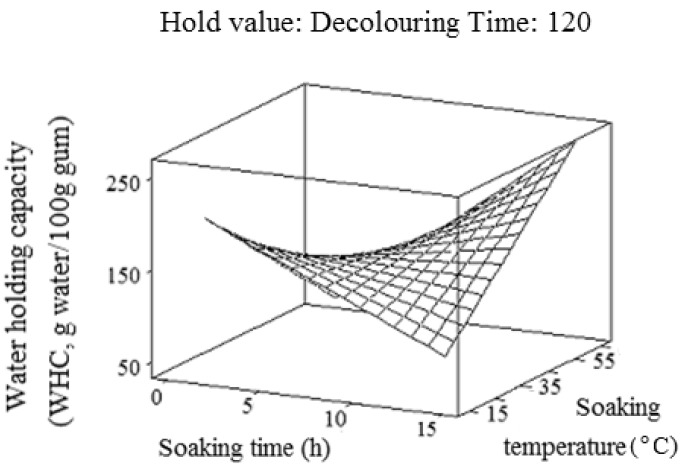
Response surface plot for explaining the variation of water-holding capacity (WHC) as a function of soaking time and temperature.

The results indicated that WHC varied from 94.1 to 218.5 (g water/100g gum) (Figure 4). As stated by Kinsella [35], WHC depends on the interaction between water and compound, amount of hydration positions or active side (OH) and environmental condition. 

The wide range of WHC could be explained by the significant effect of extraction variables on the chemical composition (*i.e*., carbohydrate profile and protein fraction) of crude durian seed gum. Janaki and Sashidhar [36] also illustrated that this variation might be attributed to the differences in the composition of the gum. The variation of WHC could be also explained by the conformational structure of the gum. As explained by Hermansson *et al*. [37], a fine, uniform structure with numerous small pores would probably result in more absorptive capacity and better water retention than a coarse structure with large pores. The results showed that WHC of chemically-extracted durian seed gum was found to be lower than that of reported for fenugreek gum (270 g water/100 g gum) [38] and *Ziziphus mauritiana* Lam gum (>500 g water/100 g dry matter) [39]. Conversely, it was higher than WHC reported for *Vigna radiate* gum (63 g water/100 g gum) [40], karaya gum (67–111 g water/100 g gum) [13], and rhizome flour of lotus (2.56 ± 0.05%) [34]. Thongsombat *et al*. [41] also reported a relatively low WHC (90 g water/100 g gum) for pectin. Figuerola *et al*. [42] compared WHC of apple, grapefruit, lemon and orange ﬁbres. They reported that the capacity of water-holding of fiber concentrates varied between 162 and 226 (g/100 g dry matter). Larrauri *et al*. [43] also reported the relatively high WHC (210 g/100 g dry matter) in pineapple fiber. These values are close to the WHC of crude durian seed gum. 

In our preliminary study, it was observed that the rate of water-holding decreased as the absorption process continued (data not shown). This was also reported in the previous study [44]. This could be due to the movement of water into capillary and intermicellar spaces, thus filling the free spaces [44]. This phenomenon decreases the driving force for the water absorption, thus reducing the absorption rate. The seed absorbs moisture, swells up, and some water-soluble nutrients leach into the soaked water by simple diffusion during the soaking process. This swelling behavior depends on the nature and ratio of the matrix and solvent, the soaking temperature and the length of the soaking time [45]. Table 1 demonstrated that the interaction effects of the soaking time and temperature positively influenced the water-holding capacity (WHC, Y_4_). On the other hand, WHC (Y_4_) was negatively proportional to the main effects of the soaking time and temperature (Table 1). The results indicated that only the main and interaction effects of the soaking time and temperature exhibited a significant (*p* < 0.05) effect on WHC. Conversely, the decolouring time did not show any significant (*p* > 0.05) effect on WHC (Table 2). Therefore, it should be removed from the final reduced model. The interaction effect of the soaking temperature and soaking time showed the most significant (*p* < 0.05) effect on WHC; while the main effect of the soaking time had the least significant (*p* < 0.05) effect on WHC of chemically-extracted durian seed gum (Table 2). 

### 2.5. Oil-Holding Capacity (OHC)

**Figure 5:**
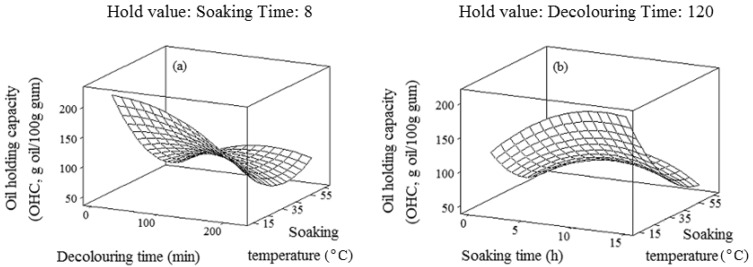
Response surface plots for explaining the variation of oil-holding capacity (OHC) as a function of (**a**) decolouring time and soaking temperature and (**b**) soaking time and temperature.

In the present study, oil-holding capacity (OHC) of chemically-extracted durian seed gum varied from 43.7 to 128.8 (g oil/100 g gum) depending on the extraction condition (Figure 5). This value was almost similar to the OHC reported by Galla and Dubasi [13] for karaya gum (81–114 g oil/100 g gum) and higher than the OHC reported by previous researchers [44] for rhizome flour of lotus (2.03 ± 0.25%). Figuerola *et al*. [44] also reported that OHC of dietary fibers from Liberty apple and Valencia orange were 60 (g oil/100 g dry matter) and 181 (g oil/100 g dry matter), respectively. OHC of hydrocolloids depends on the chemical and conformational structure of hydrocolloid, e.g., the ratio and position of hydrophobic to hydrophilic groups present in the hydrocolloid structure. High OHC is related to enhance the hydrophobic character [34]. The chemical extraction resulting in lower content of the hydrophobic fraction leads to a reduced gum OHC. As shown in Table 1, OHC was positively proportional to the main effect of the decolouring time and soaking time as well as the interaction effect of the decolouring time and soaking temperature (Table 1), whereas, OHC (Y_5_) was negatively related to the main effect of the soaking temperature as well as the interaction effect of the soaking time with the decolouring time and soaking temperature (Table 1). Kinsella [46] illustrated that the mechanism of oil-holding was mostly attributed to the physical entrapment of oil by the non-polar chains of protein. Higher oil-holding capacity might also be due to the presence of non-polar amino acids [47]. Table 2 demonstrated that the soaking time and decolouring time exhibited the most and least significant (*p* < 0.05) effect on the oil-holding capacity. All extraction variables except for single effect of the decolouring time were significantly (*p* < 0.05) fitted in the final reduced model. Among all functional properties, the chemical extraction variables showed the most significant effect on the oil-holding capacity of crude durian seed gum (Table 2). Figure 5 showed that OHC increased with prolonging the soaking time. The chemical extraction at low soaking temperature for the short decolouring time also led to increase OHC. 

## 3. Experimental

### 3.1. Chemicals and Standards

### 3.2. Chemical Extraction of Crude Durian Seed Gum

**Figure 6:**
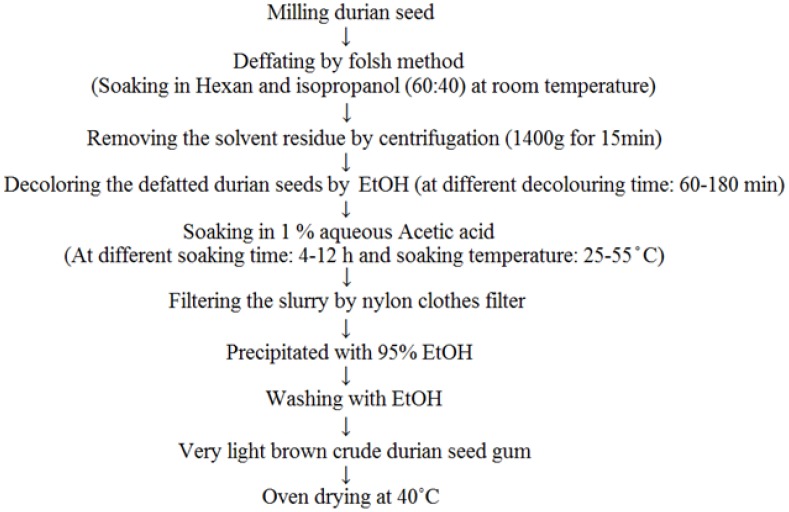
Flow chart of chemical extraction of durian seed gum [48].

Chemical extraction was performed according to the method described by Singh *et al.* [48] with the minor modifications. The successive steps of defatting, decolouring, solvent soaking, gum dissolution, centrifugation, and precipitation were considered for the chemical extraction (Figure 6). Durian seeds were washed and chopped into small pieces. Then, they were air dried using air circulation before milling into flour. Cold extraction was used to extract the oil from the durian seed flour in order to avoid any thermal degradation. A preliminary study showed that the solvent mixture containing hexane and isopropanol (60:40) was the most efficient solvent for the defatting process among all studied solvents (*i.e*., petroleum ether, hexane, isopropanol and ethanol), so the defatting process was carried out using this solvent mixture at room temperature (25 ± 1 °C). The solvent residue was removed by centrifugation at 1,400 g for 15 min (Avanti J-25 Centrifuge, Beckman Coulter GmbH, Krefeld, Germany). Then, defatted-durian seed flour (1 kg) was exhaustively decolored using ethanol at different decolouring time (60–180 min). The decolourized seed flour was vacuum filtered and then soaked in 1% aqueous acetic acid for different soaking times (4–12 h) and at different temperatures (25–55 °C). Then, the slurry was filtered with nylon cloth filter. The filtrate was precipitated with 95% ethanol, and the precipitated slurry was washed three times using absolute ethanol (99.9%) to produce a very light brown amorphous crude gum. The crude gum was collected and oven dried at 40 °C.

### 3.3. Analytical Tests

#### 3.3.1. Volume-Weighted Means (D [4,3])

The volume-weighted mean (D [4,3] or average particle size) of durian seed gum in the aqueous system was measured by a particle size analyzer equipped with an accessory Hydro 2000S (Malvern Mastersizer 2000, Malvern Instrument Limited, Worcestershire, UK). In this experiment, 0.5 g of crude durian seed gum was dispersed in distilled water (0.5% w/w). The Malvern Mastersizer 2000, which is considered a spatial sampling device, correlates with the phenomena that a particle passing through the laser beam can cause the laser light to scatter in many angles dependent on the particle diameter. Particle characteristics were computed automatically from a compressed range [24]. A few drops of the gum dispersion (0.5% w⁄w) were introduced into the diluting chamber of the instrument until the beam obstruction level fell within the optimum range displayed on the screen. The dilution chamber was an integrated ultrasonic water bath equipped with a propeller-type stirrer. It exposes the sample to a shear force high enough to ensure breakage of flocks and aggregates and hence uniform distribution of droplets. The measurement readings of the volume-weighted mean were reported as average of four individual replications for each sample. The volume-weighted mean diameter is estimated by the following equation [24,49]:


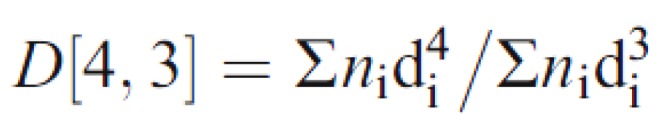


where n_i_ is the number of particles with diameter d_i_ [24,49]. 

#### 3.3.2. Span

Particle size distribution (PSD) was determined by measuring the span [50]. The seed gum solution (0.5% w⁄w) was prepared by dispersing crude durian seed gum in deionized water (refractive index, RI = 1.33 and absorption value = 0.1) using mechanical stirring at 500 rpm for 1 h at 25 °C. Subsequently, the seed gum solution (0.5% w⁄w) was hydrated for 24 h prior to analysis. Then, span was measured by using the laser diffraction particle analyzer (Mastersizer 2000) based on the following equation:


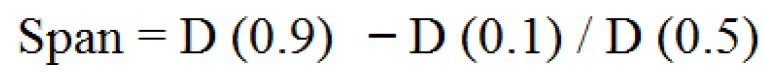


where D (v, 0.1), D (v, 0.5) and D (v, 0.9) are diameters at 10%, 50% and 90% cumulative volume, respectively. The span measurement (*i.e*., droplet size distribution) was carried out immediately after preparing the dispersion (0.5% w/w) in four replications for each sample [49]. 

#### 3.3.3. Solubility

The solubility was determined according to the previous researchers [51], with minor modification. One g of seed gum powder was added to 100 mL of distilled water. Then, the mixture was agitated with mechanical stirring at elevated temperature (80 °C) for 30 min. The solubility of samples was measured at this elevated temperature (80 °C). The gum solution was then centrifuged at 6,000 g for 30 min to remove the insoluble material. The supernatant was transferred to disposable Petri dishes and oven dried at 105 °C for 24 h until constant weight. The percent solubility was calculated by the weight difference and expressed in dry basis. The determination of solubility was carried out in triplicate. Therefore, the average of three measurements was considered for further data analysis.

#### 3.3.4. Water- and Oil-Holding Capacity

Water-holding capacity (WHC) for crude durian seed gum was determined as described in the literature [13,52] with minor modifications. One g of crude durian seed gum was suspended in 10 mL of distilled water, vortexed for 2 min and then centrifuged with a refrigerated centrifuge 3–18 K (Sartorius, Sigma 3-18, Gottingen, Germany) at 3,000 g for 30 min. The free water was decanted and the water absorbed by the samples was expressed as grams of water absorbed per 100 g of seed gum. Oil-holding capacity (OHC) was also determined by dispersing 1 g of crude durian seed gum in 10 mL of refined sunflower oil, and repeated the experiment. It was expressed as grams of oil absorbed per 100 g of seed gum [13]. The measurements were performed in triplicate for each sample. In the current study, WHC and OHC were calculated based on the following equations: 


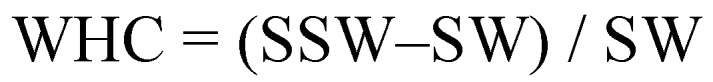



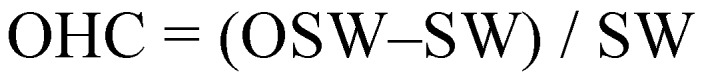


where SSW, OSW and SW are the water swollen sample weight, oil-adsorbed sample weight and sample weight, respectively [52]. 

### 3.4. Experimental Design and Data Analysis

A three-factor central composite design was used to investigate the effect of chemical extraction conditions (*x*_1_, decolouring time; *x*_2_ soaking time; and *x*_3_, soaking temperature) on physical and functional properties (span, volume weighted mean, solubility (80 °C), WHC and OHC respectively) (Table 3). Twenty experimental treatments were assigned based on the CCD with three independent variables at five levels of each variable (Table 3).

**Table 3:** Matrix of the central composite design (CCD) for chemical extraction.

Runs	Blocks	Decolouring time (x_1_, min)	Soaking time (x_2_, h)	Soaking temperature (x_3_, °C)
1 *	1	120.0	8.0	40.0
2	1	60.0	4.0	25.0
3	1	180.0	12.0	25.0
4	1	60.0	12.0	55.0
5	1	180.0	4.0	55.0
6 *	1	120.0	8.0	40.0
7	2	180.0	12.0	55.0
8	2	60.0	4.0	55.0
9 *	2	120.0	8.0	40.0
10	2	60.0	12.0	25.0
11	2	180.0	4.0	25.0
12 *	2	120.0	8.0	40.0
13	3	120.0	14.5	40.0
14	3	218.0	8.0	40.0
15 *	3	120.0	8.0	40.0
16	3	120.0	8.0	64.5
17	3	120.0	8.0	15.5
18 *	3	120.0	8.0	40.0
19	3	22.0	8.0	40.0
20	3	120.0	1.5	40.0

Note: (*), centre point.

Extraction conditions ranges studied were decolouring time (60–180 min), soaking time (4–12 h) and soaking temperature (25–55 °C). It should be reminded that all eight factorial points and six centre points fall inside the range of chemical extraction variables. The presence of six star points outside the studied ranges can help the researchers to find out the possible unexpected changes of the response which may occur beyond the studied ranges [53]. The changes of functional properties of gum were predicted according to the following equation:


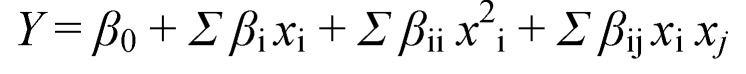


where *Y* is response calculated by the model, *β*_0_ is a constant, *β*_i_, *β*_ii_ and *β*_ij_ are linear, squared and interaction coefficient, respectively [54]. The final regression model was only fitted to those statistically significant (*p <* 0*.*05) terms. However, some insignificance variables were still kept in the final reduced model due to the presence of significant (*p* < 0.05) quadratic or interaction term containing this variable [55]. The adequacy of the model was determined using model analysis, lack of fit and coefficient of determination (R^2^) [56]. Minitab version 15 (Minitab Inc., State College, PA, USA) was used for the creation of the experimental design and data analysis. 

## 4. Conclusions

In the current study, the effect of three extraction variables on the physicochemical and functional properties of durian seed gum was investigated. The current study revealed that the soaking process had a more significant impact than the decolouring process on the physicochemical and functional properties of durian seed gum. This might be explained by the considerable impact of the soaking process on the quantity and quality of impurities present in the crude gum structure. The current work reveals that the chemical extraction under a shorter decolouring and soaking time results in narrower particle size distribution. The present study suggests that the soaking time should be considered as the most critical extraction variable affecting the functional properties of the chemically-extracted durian seed gum. The crude durian seed gum showed a relatively low solubility and WHC. This might be due to the presence of water insoluble impurities and large particles. Therefore, further modification of the process is recommended to improve the solubility, water adsorption capacity and other functional properties of crude durian seed gum. 

## References

[1] Ibañez M.C., Ferrero C. (2003). Extraction and characterization of the hydrocolloid from *Prosopis flexuosa* DC seeds. Food Res. Int..

[2] Rana V., Rai P., Tiwary A.K., Singh R.S., Kennedy J.F., Knill C.J. (2011). Modified gums: Approaches and applications in drug delivery. Carbohydr. Polym..

[3] Jun T.Y., Arumugam S.D., Abdul Latip N.H., Abdullah A.M., Abdul Latif P. (2010). Effect of activation temperature and heating duration on physical characteristics of activated carbon prepared from agriculture waste. Environ. Asia.

[4] Glicksman M. (1969). A Series of Monographs: Gum Technology in the Food Industry.

[5] Vardhanabhuti B., Ikeda S. (2006). Isolation and characterization of hydrocolloids from monoi (*Cissampelos pareira*) leaves. Food Hydrocol..

[6] Amin A.M., Ahmad A.S., Yin Y.Y., Yahya N., Ibrahim N. (2007). Extraction, purification and characterization of durian (*Durio zibethinus*) seed gum. Food Hydrocol..

[7] Somboonpanyakul P., Wang Q., Cui W., Barbut S., Jantawat P. (2006). Malva nut gum. (Part I): Extraction and physicochemical characterization. Carbohydr. Polym..

[8] Mohammadzadeh Milani J., Emam-Djomeh Z., Rezaee K., Safari M., Ganbarzadeh B., Gunasekaran S. (2007). Extraction and physicochemical properties of Barijeh (*Ferula galbaniflua*) gum. Int. J. Agric. Biol..

[9] Ho C.H.L., Cacacea J.E., Mazza G. (2007). Extraction of lignans, proteins and carbohydrates from flaxseed meal with pressurized low polarity water. LWT Food Sci. Technol..

[10] Kawamura Y. (2008). Food Safety and Quality:Carob Bean Gum Chemical and Technical Assessment(CTA) for the Joint FAO/WHO Expert Committee on Food Additives (JECFA). Food and Agriculture Organization of the United State.

[11] Chaires-Martínez L., Salazar-Montoya J.A., Ramos-Ramírez E.G. (2008). Physicochemical and functional characterization of the galactomannan obtained from mesquite seeds (*Prosopis pallida*). Eur. Food Res. Technol..

[12] Razavi S.M.A., Mortazavi S.A., Matia-Merino L., Hosseini-Parvar S.H., Motamedzadegan A., Khanipour E. (2009). Optimisation study of gum extraction from Basil seeds (*Ocimum basilicum* L.). Int. J. Food Sci. Technol..

[13] Galla N.R., Dubasi G.R. (2010). Chemical and functional characterization of gum karaya (*Sterculia urens* L.) seed meal. Food Hydrocol..

[14] Mirhosseini H., Tan C.P., Aghlara A., Hamid N.S.A., Yusof S., Boo H.C. (2008). Influence of pectin and CMC on physical stability, turbidity loss rate, cloudiness and flavor release of orange beverage emulsion during storage. Carbohydr. Polym..

[15] Mirhosseini H., Tan C.P., Hamid N.S.A., Yusof S. (2009). Characterization of the influence of main emulsion components on cloudiness, size index, conductivity and emulsion stability of orange beverage emulsion using response surface methodology. Food Hydrocol..

[16] Mirhosseini H., Tan C.P., Naghshineh M. (2010). Influence of pectin and CMC content on physicochemical properties of orange beverage emulsion. J. Food Agric. Environ..

[17] Mirhosseini H., Tan C.P. (2010). Effect of various hydrocolloids on physicochemical characteristics of orange beverage emulsion. J. Food Agric. Environ..

[18] Mirhosseini H., Tan C.P., Taherian A.R. (2008). Effect of glycerol and vegetable oil on physicochemical properties of Arabic gum-based beverage emulsion. Eur. Food Res. Technol..

[19] Mirhosseini H., Tan C.P., Hamid N.S.A., Yusof S. (2009). Characterization of the main emulsion components on cloudiness, size index, conductivity and emulsion stability of orange beverage emulsion using response surface methodology. Food Hydrocol..

[20] Tabatabaee Amid B., Mirhosseini H. (2012). Optimization of aqueous extraction of gum from Durian (*Durio zibethinus*) seed: A potential, low cost source of hydrocolloid. Food Chem..

[21] Chew N.Y.K., Chan H.K. (2002). Effect of powder polydispersity on Aerosol Generation. J. Pharm. Sci..

[22] (1999). Malvern Instruments Operators Guide, Man. 0247 Issue 2.0.

[23] Sarkar A., Rano R., Mishra K.K., Sinha I.N. (2005). Particle size distribution proﬁle of some Indian ﬂy ash: A comparative study to assess their possible uses. Fuel Proc. Technol..

[24] Ghorbani Gorji E., Mohammadifar M.A., Ezzatpanah H. (2011). Influence of gum tragacanth, Astragalus gossypinus, addition on stability of non-fat Doogh, an Iranian fermented milk drink.. Int. J. Dairy Technol..

[25] Laaman T.R. (2011). Hydrocolloids: Fifteen Practical Tips. Hydrocolloids in Food Processing.

[26] Maier H., Anderson M., Karl C., Magnuson K., Whistler R.L., Whistler R.L., BeMiller J.N. (1933). Guar, Locust Bean, Tara and Fenugreek Gums. Industrial Gums, Polysaccharides and their Derivates.

[27] Koocheki A., Taherian A.R., Bostan A. (2011). Studies on the steady shear flow behaviour and functional properties of *Lepidium perfoliatum* seed gum. Food Res. Int..

[28] Ophardt C.E. Temperature and Pressure Effects on Solubility. Virtual Chembook.

[29] Knudsen K.E.B. Dietary Fibre in Nutrition and Health of Piglets. http://www.pig333.com/nutrition/dietary-fibre-in-nutrition-and-health-of-piglets_1256/.

[30] Singh U. (2001). Functional properties of grain legume flours. J. Food Sci. Technol..

[31] Simas-Tosin F.F., Barraza R.R., Petkowicz C.L.O., Silveira J.L.M., Sassaki G.L., Santos E.M.R., Gorin P.A.J., Iacomini M. (2010). Rheological and structural characteristics of peach tree gum exudates. Food Hydrocol..

[32] Torio M.A.O., Saez J., Merc F.E. (2006). Physicochemical characterization of galactomannan from sugar palm (*Arenga saccharifera* Labill.) endosperm at different stages of nut maturit. Philippine J. Sci..

[33] Grigelmo-Miguel N., Martin-Belloso O. (1999). Characterization of dietary fibre from orange juice extraction. Food Res. Int..

[34] Shad M.A., Nawaz H., Hussain M., Yousuf B. (2011). Proximate composition and functional properties of rhizomes of lotus (*Nelumbo nucifera*) from Punjab, Pakistan. Pakistan J. Bot..

[35] Kinsella J.E., Fox P.F., Condon J.J. (1982). Relationships between Structural and Functional Properties of Food Proteins. Food Protein.

[36] Janaki B., Sashidhar R.B. (1998). Physicochemical analysis of gum Kondagogu (*Cochlospermum gossypium*): A potential food additive. Food Chem..

[37] Hermansson A.M., Harbitz O., Langton M. (1986). Formation of two types of bovine myosin gels. J. Sci. Food Agric..

[38] Chang Y.H., Cui S.W., Roberts K.T., Ng P.K.W., Wang Q. (2011). Evaluation of extrusion-modified fenugreek gum. Food Hydrocol..

[39] Thanatcha R., Pranee A. (2011). Extraction and characterization of mucilage in *Ziziphus mauritiana* Lam. Int. Food Res. J..

[40] Asha D., Shastri P.N. (2004). Changes in structure, fat binding and water absorption of starch during roasting of wheat and legume flour. J. Food Sci. Technol..

[41] Thongsombat W., Sirichote A., Chanthachum S. (2007). The production of guava juice fortified with dietary fiber. Songklanakarin J. Sci. Technol..

[42] Figuerola F., Hurtado M.L, Estévez A.M., Chiffelle I., Asenjo F.  (2005). Fibre concentrates from apple pomace and citrus peel as potential fibre sources for food enrichment. Food Chem..

[43] Larrauri J.A., Rupérez P., Bravo L., Saura-Calixto F. (1996). High dietary ﬁbre powders from orange and lime peels, associated polyphenols and antioxidant capacity. Food Res. Int..

[44] Abu-Ghannam N., McKenna B. (1997). The application of peleg’s equation to model water absorption during the soaking of red kidney beans (*Phaseolus vulgaris* L.). J. Food Eng..

[45] Bressani R., Benavides V., Acevedo E., Oritiz M.A. (1990). Changes in selected nutrient content and in protein quality of common and quality protein maize during torilla preparation. Cereal Chem..

[46] Kinsella J.E. (1976). Functional properties of proteins in foods: A survey. Crit. Rev. Food Sci. Nut..

[47] Lazos E.S. (1992). Certain functional properties of defatted pumpkin seed flour. Plant Food Hum. Nut..

[48] Singh V., Singh S.K., Maurya S. (2010). Microwave induced poly (acrylic acid) modification of *Cassia javanica* seed gum for efficient Hg (II) removal from solution. Chem. Eng. J..

[49] Kováčová R., Synytsya A., Štětina J. (2009). Characterisation of whey proteins-pectin interaction in relation to emulsifying properties of whey proteins. Czech J. Food Sci..

[50] León-Martínez F.M., Rodríguez-Ramírez J., Medina-Torres L.L., Méndez Lagunas L.L., Bernad-Bernad M.J. (2011). Effects of drying conditions on the rheological properties of reconstituted mucilage solutions (*Opuntia ficus-indica*). Carbohydr. Polym..

[51] Dakia P.A., Blecker C., Roberta C., Watheleta B., Paquota M. (2008). Composition and physicochemical properties of locust bean gum extracted from whole seeds by acid or water dehulling pre-treatment. Food Hydrocol..

[52] Sciarini L.S., Maldonado F., Ribotta P.D., Pérez G.T., León A.E. (2009). Chemical composition and functional properties of *Gleditsia triacanthos* gum. Food Hydrocol..

[53] Montgomery D.C. (2001). Design and Analysis of Experiments.

[54] Mirhosseini H., Tan C.P., Taherian A.R., Boo H.C. (2009). Modeling the physicochemical properties of orange beverage emulsion as function of main emulsion components using response surface methodology. Carbohydr. Polym..

[55] Mirhosseini H., Tan C.P. (2010). Discrimination of orange beverage emulsions with different formulations using multivariate analysis. J. Sci. Food Agric..

[56] Joglekar A.M., May A.T. (1987). Product excellence through design of experiments. Cereal Food. World.

